# Where do we stand with IPF treatment?

**DOI:** 10.1186/1465-9921-14-S1-S7

**Published:** 2013-04-16

**Authors:** C Albera, C Ferrero, E Rindone, S Zanotto, E Rizza

**Affiliations:** 1 Università diTorino, Facoltà di Medicina e Chirurgia San Luigi Gonzaga Dipartimento di Scienze Cliniche e Biologiche Scuola di Specializzazione in Malattie dell'Apparato Respiratorio: Direzione Corso di Laurea in Infermieristica, Sedi di Orbassano e Cuneo, Torino, Italy

**Keywords:** Idiopathic Pulmonary Fibrosis (IPF), Panther, pirfenidone, N-acetylcysteine (NAC)

## Abstract

Despite receiving ‘weak no’ recommendations in the updated guidelines on treating patients with Idiopathic Pulmonary Fibrosis (IPF), two key treatment options are pirfenidone and N-acetylcysteine (NAC), and both are used in clinical practice. The efficacy of pirfenidone is supported by a number of Phase III trials as well as a Cochrane meta-analysis. Tolerability data are also provided by clinical trials and a long-term extension phase of these studies. Pirfenidone is approved in Europe for the treatment of patients with mild-to-moderate IPF. NAC-based therapy has no such approval, but is commonly used to treat patients. A Phase III trial suggested some benefit of the NAC, prednisone and azathioprine regimen for IPF patients, but the study had many limitations. A further study to investigate this regimen, compared with a placebo alone arm, was recently stopped due to increased mortality in the triple-therapy arm. Discussion of these data and recent findings highlight the importance of a further update to the existing guidelines, so that IPF specialists can provide the most up-to-date advice and treatment to patients in clinical practice.

## Introduction

The 2011 guideline update on the treatment of Idiopathic Pulmonary Fibrosis (IPF) unfortunately did not strongly or weakly recommend any pharmacological agent for use. [1] A strong recommendation was made against the use of corticosteroids, colchicine, cyclosporine A, interferon γ 1b, bosentan and etanercept. Treatments given ‘weak no’ recommendations were pirfenidone, anticoagulation, N-acetylcysteine (NAC) monotherapy, and triple-therapy with NAC, azathioprine and prednisone. For the latter group, this has meant that they are not to be considered suitable for use in the majority of patients but may be a reasonable treatment choice for a minority of patients. Pirfenidone received this recommendation despite data from a Cochrane meta-analysis [2] supporting its efficacy, although at the time the recommendations were finalised results from two out of four trials [3-5] used in the Cochrane meta-analysis were yet to be published. Of all medications studied for the treatment of IPF, pirfenidone currently has the highest grade of evidence to support its efficacy and is the only medication approved for use in this indication. Regardless of the 2011 recommendations, triple-therapy with NAC, azathioprine and prednisone is considered standard treatment by some treating physicians in the field of IPF. This is mainly due to data coming from the IFIGENIA study, [6] which despite its numerous limitations suggested preservation of lung function in patients with IPF. A further study [7] of this triple-therapy regimen was underway, but recently the triple-therapy arm of the study was stopped following a planned interim analysis that revealed an increased rate of death and hospitalisation compared with placebo. This regimen is still used in practice, but this is off-label treatment. The impact of data from these studies on the current treatment paradigm will be discussed, along with issues that remain to be addressed by the updated guidelines and the IPF community as a whole.

## Data from key studies of NAC-based triple-therapy

Data from the IFIGENIA Study provided support for the use of NAC plus prednisone and azathioprine to treat IPF patients. Patients were treated with high-dose NAC (600 mg TID) plus prednisone and azathioprine. The primary endpoint of the study was change in vital capacity. The NAC-based triple-therapy was found to significantly reduce decline in VC after one year of treatment. However, this study had several limitations. Not all patients included in the study were included in the Intention-To-Treat (ITT) analysis. Furthermore, as all patients were treated with prednisone plus azathioprine, this did not allow a direct comparison with patients treated only with placebo, i.e. a placebo alone group. In view of the drop-out rate from this study of approximately 30% (including deaths) questions were raised regarding the clinical relevance and robustness of the treatment effect. [8] Despite these issues, the NAC, prednisone and azathioprine treatment regimen is commonly used in clinical practice. Subgroup analyses from the study confirmed the favourable effects of NAC on lung function, and patients with less advanced disease at baseline appeared to be more responsive to NAC treatment. [9]

The PANTHER-IPF Study was designed to determine whether NAC-based triple-therapy could slow disease progression and improve lung function in patients with moderate IPF. In addition, this study compared the triple-therapy regimen with NAC monotherapy and placebo alone. In 2011, the NIH announced that the triple-therapy arm of the trial had been stopped due to increased mortality observed in this treatment group. [10] A recently published statement by five European experts has attempted to provide some urgently needed guidance on the implications of these findings to routine clinical practice. [11] Meanwhile, the other two treatment arms have continued, and some data from the terminated NAC, prednisone and azathioprine arm have just been published. [7] Currently, if we have patients being treated with this regimen we should discuss any concerns and decide the next steps along with the patient in question.

## Data from key studies of pirfenidone

Three Phase III studies have investigated the efficacy and safety of pirfenidone in patients with IPF. The first was a Japanese study in which patients were randomised to pirfenidone or placebo. [4] The primary endpoint was change in vital capacity (VC) from baseline to 52 weeks. Statistically significant differences were observed between the pirfenidone and placebo groups for the primary endpoint along with secondary endpoints. Pirfenidone was associated with a 44% reduction in the VC decline compared with placebo (p=0.0416), and with a significant increase in progression-free survival (p=0.0280). Pirfenidone was usually well tolerated. Data from this study led to the approval of pirfenidone in Japan in 2008.

The multinational CAPACITY trials (CAPACTY 1 and 2) investigated the efficacy of pirfenidone in two large, concurrent Phase III trials. Patients with mild-to-moderate IPF were randomised to treatment with pirfenidone or placebo. The primary endpoint of both studies was changed from baseline in percentage predicted FVC at week 72. Data from the CAPACITY 1 Study, and the pooled analysis of both studies supported the efficacy of pirfenidone. The primary endpoint however was not met in CAPACITY 2. Meta-analyses of data by Spagnolo et al. for the Cochrane Collaboration [2] confirmed the treatment effect of pirfenidone. Data from the two Japanese studies [3,4] showed a statistically significant difference was observed in terms of decline in VC in favour of pirfenidone. PFS data from the Phase III study by Taniguchi et al. was combined with data from the CAPACITY Studies. The overall result of this meta-analysis showed that pirfenidone reduced the risk of disease progression by 30% in patients with IPF. [2]

A recent review by Germany’s Federal Joint Committee granted the additional benefit of pirfenidone in adults with mild-to-moderate IPF, [12] despite an initial proposal from the Institute for Quality and Efficiency in Health Care (IQWiG) [13] that concluded ‘no proven benefit’. The additional benefit was classified as Stage 4 (a non-quantifiable benefit) in the rating system established under German pharmaceutical law. This means that pirfenidone is considered to have an additional benefit, which will likely be defined in the future via experience in daily clinical use or clinical studies.

In terms of safety, pirfenidone was usually well tolerated at the standard 2403 mg/day dose in the CAPACITY Studies. There was no significant difference in the number of patients experiencing serious treatment-emergent adverse events between the pirfenidone and placebo groups (33% and 31% respectively; pooled CAPACITY data). The most common adverse events being gastrointestinal, skin disorders and dizziness. These are consistent with the known safety profile of pirfenidone and were generally mild-to-moderate in severity. The extension phase of the CAPACITY Studies (RECAP) aimed to assess the safety of pirfenidone beyond the duration of these Phase III Studies. At Week 72 of this extension phase, patients had been treated with pirfenidone for a mean duration of 2.9 years, with 114 patients treated at the full dose for at least three years. Safety data from the RECAP extension phase confirm the tolerability of pirfenidone. [14] Common adverse events observed were very similar to those observed in the CAPACITY Studies, and were generally mild-to-moderate in severity. Rash/photosensitivity occurred in fewer patients from the RECAP Extension phase than in the CAPACITY Studies (20% vs 44%). Interestingly, rash or photosensitivity was more common among patients initiating treatment with pirfenidone compared with those who were on continued treatment (28% vs 12%). [13] These data provide further important information on treatment with pirfenidone and demonstrate its tolerability.

## Conclusions

Based on the evidence available to date, pirfenidone is proven effective and generally well tolerated in the treatment of patients with IPF. In addition, the tolerability of pirfenidone has been demonstrated in clinical trials and a long-term extension phase. However, an inherent issue with approval of pirfenidone in Europe is that it is indicated for patients with mild-to-moderate disease. The approval in these patients was based on the functional criteria used in the CAPACITY studies, which were: FVC ≥50% of predicted value, DL_CO_ ≥35% of predicted value, and a 6MWT distance of ≥150 m. [4] In clinical practice it is therefore a challenge to define patients with mild-to-moderate disease, particularly in the absence of any current staging or classification system. It is essential that we determine the best way to classify our patients with IPF so that we can give them the most appropriate treatment based on their disease stage. The need for an update to the 2011 ATS/ERS/JRS/ALAT recommendations document is highlighted by the fact that four published studies [3-5] and a Cochrane meta-analysis [2] support the patient benefit associated with pirfenidone treatment, along with further safety data from the long-term RECAP Study. [14] In addition, NAC-based therapy is commonly used to treat IPF patients, and we await the results from the PANTHER-IPF study regarding the NAC monotherapy arm.

## Competing interests

Dr Carlo Albera has served as investigator in clinical trials, consultant, speaker, Steering Committee or Scientific Advisory Board member for: Actelion, Almirall, Aptalis, Bayer, Centocor, Eli-Lilly, GSK, InterMune, Inc.

## Authors’ contributions

All the authors contributed equally.

## Declarations

This article has been published as part of *Respiratory Research* Volume 14 Supplement 1, 2013:IPF in 2011 – Key updates on guidelines and therapeutics. The full contents of the supplement are available online at http://respiratory-research.com/supplements/14/S1. Publication of this supplement was supported by IntraMed Communications with funding from InterMune, AG. InterMune is the manufacturer of pirfenidone, a product mentioned in this article. The supplement originates from presentations given at the “AIR Event: Advancing IPF Research. Working together to translate IPF research into practice” held in Berlin in November 2011. The publication was proposed by IntraMed Communications and developed in consultation with the journal. All articles in the supplement have undergone the journal’s standard peer review process.
